# Efficient americium separation

**DOI:** 10.1038/s44172-022-00008-5

**Published:** 2022-05-26

**Authors:** Mengying Su

**Affiliations:** Communications Engineering, https://www.nature.com/commseng

## Abstract

A chemical strategy to separate troublesome americium from lanthanides could help reduce the radiological hazards of spent nuclear fuel and create opportunities for waste re-processing.

**Figure:**
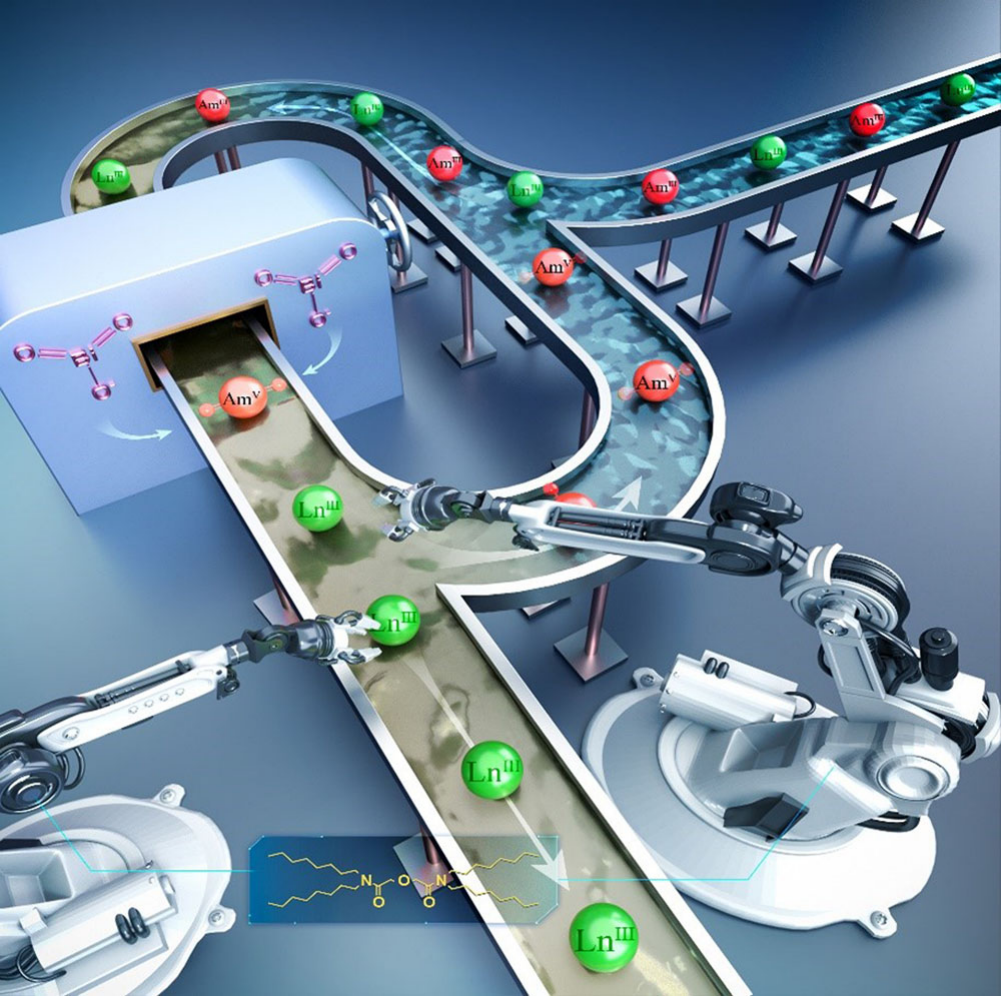
Chao Xu

Nuclear power could provide stable energy supplies for countries with limited natural energy resources and also offer a potential route towards decarbonization. However, the ecological and toxicological implications of nuclear waste remain an ongoing challenge. Americium (Am) is a troublesome radioactive element found in spent nuclear fuel. Its separation from other fission products (such as lanthanide elements) could enhance repository storage capacity as well as minimize the long-term health and ecological hazards of nuclear waste. However, in particular, it has proven a challenge to separate Am from lanthanides because they exhibit similar chemical properties. Research recently published in the *Journal of the American Chemical Society* describes a new route to separate Am from lanthanides.

Previous reports have suggested that the difference in oxidizability between lanthanides and Am could be harnessed for separations via solvent extraction: Am can be oxidized to higher oxidation states (V) and (VI) in strong oxidizing solutions while lanthanides will not^1^. Applying the approach for separations would require maintaining higher oxidation states over sufficiently long timeframes. However, both Am (V) and Am (VI) are sensitive to organic solvents used in biphasic solvent extraction processes and they rapidly reduce to Am (III) once in contact with the organic reagents^2^. The rapid reduction to lower oxidation state creates problems, limiting the separation to strict processing environments: high-valent Am samples must be freshly prepared and the reaction time is limited to only a few seconds.

Zhipeng Wang, Jun-Bo Lu and colleagues from Tsinghua University designed a strategy to generate and stabilize aqueous Am (V) ions using a modified organic solvent. The researchers created a unique redox environment by loading highly oxidative Bi (V) (bismuthic acid) species into an organic solvent of n-dodecane in the presence of a tridentate ligand. When an aqueous acidic solution containing Am (III) species was then placed in contact with the Bi (V)-modified organic solvent, the researchers observed the presence Am (V) ions in the aqueous phase, with 96% of the Am (V) stable for longer than 2 h. Further experimental and theoretical investigations suggested that Am (V) was generated in the organic phase and back-extracted into the acidic aqueous phase. In contrast, most of the lanthanides remained at a lower oxidation state, and separated into the organic solvent. The separation of high-concentration Am from lanthanides was also demonstrated by directly treating a simulated waste feed solution. When an aqueous solution containing both lanthanides and Am (III) was placed in contact with the Bi (V)-containing organic solution, lanthanide ions entered into the organic phase while stable Am (V) species were found in the aqueous phase. Am was separated from selected lanthanide ions (La, Ce, Pr, Nd, Sm, Eu, and Gd) with a mean average separation factor of 46,000, while an even higher separation factor of 137,000 was obtained in the Ce/Am separation. As far as the researchers are aware, the observed results outperform all previous separations using an oxidation state control approach.

Corresponding author Chao Xu described the demands of working in this field. “In this work, we have to conduct experiments involving millimolar levels of highly radioactive ^241^Am. Although we have proper personal protective equipment and a fully qualified radiological lab, manipulating such high level of ^241^Am materials is always of great challenge.”

The authors suggest that the new strategy enables quantitative separation of Am and lanthanides in one single biphasic contact, and is applicable to a wide range of acid concentrations. This increases the possibility of translating the Am separation strategy for industrial scale applications in a bid to close the nuclear fuel cycle.

The original article can be found here: Wang, Z., Lu, J.B., Dong, X., Yan, Q., Feng, X., Hu, H.S., Wang, S., Chen, J., Li, J. and Xu, C., 2022. Ultra-Efficient Americium/Lanthanide Separation through Oxidation State Control. Journal of the American Chemical Society; 10.1021/jacs.2c00594.
